# Higher *RET* Gene Expression Levels Do Not Represent anAlternative *RET* Activation Mechanism in Medullary Thyroid Carcinoma

**DOI:** 10.3390/biom11101542

**Published:** 2021-10-19

**Authors:** Chiara Mulè, Raffaele Ciampi, Teresa Ramone, Alessandro Prete, Antonio Matrone, Virginia Cappagli, Liborio Torregrossa, Fulvio Basolo, Rossella Elisei, Cristina Romei

**Affiliations:** 1 Endocrine Unit, Department of Clinical and Experimental Medicine, University of Pisa, 56124 Pisa, Italy; chiaramule93@gmail.com (C.M.); raffaele.ciampi@unipi.it (R.C.); teresa.ramone@med.unipi.it (T.R.); alessandro.prete22@gmail.com (A.P.); anto.matrone@yahoo.com (A.M.); virgicap@hotmail.com (V.C.); cristina.romei@unipi.it (C.R.); 2 Department of Surgical, Medical, Molecular Pathology, University of Pisa, 56124 Pisa, Italy; libo.torregrossa@gmail.com (L.T.); fulvio.basolo@unipi.it (F.B.)

**Keywords:** *RET*, *RAS*, medullary thyroid carcinoma, mRNA expression

## Abstract

This study was designed to investigate whether *RET* (rearranged during transfection) mRNA over-expression could be considered an alternative driver event for the development of medullary thyroid carcinoma (MTC), and if different *RET* isoforms could play a role in MTC tumorigenesis. Eighty-three MTC patients, whose mutational profile was previously identified by next-generation sequencing (NGS) IONS5, were included in this study. Expression analysis was performed by the quantitative reverse transcription-polymerase chain reaction technique. *RET* expression levels were found to be significantly higher in cases with *RET* somatic mutations than in cases that were negative for *RET* somatic mutations (*p* = 0.003) as well as in cases with a somatic mutation, either in *RET* or *RAS* than in cases negative for both these mutations (*p* = 0.01). All cases were positive for the *RET51* isoform expression while only 72/83 (86.7%) were positive for *RET9* isoform expression. A statistically significant higher expression of the *RET51* isoform was found in cases positive for *RET* somatic mutation than in cases either positive for *RAS* mutation (*p* = 0.0006) or negative for both mutations (*p* = 0.001). According to our data, *RET* gene over-expression does not play a role in MTC tumorigenesis, neither as an entire gene or as an isoform. At variance, the *RET* gene, and in particular the *RET51* isoform, is expressed higher in *RET* mutated cases. On the basis of these results we can hypothesize that the overexpression of *RET*, and in particular of *RET51*, could potentiate the transforming activity of mutated *RET*, making these cases more aggressive.

## 1. Introduction

The rearranged during transfection (*RET*) proto-oncogene is localized on chromosome 10q11.2 and was first identified in 1985 based on its ability to transform NIH3T3 cells [1]. The *RET* proto-oncogene encodes for a transmembrane tyrosine kinase receptor involved in the control of cell differentiation and proliferation [2]. As well as other growth factor receptors, the *RET* gene may be involved in the development of human cancers through different activating mechanisms [3]. Activating gain of function mutations are specifically related to medullary thyroid carcinoma (MTC) [4], while *RET* gene rearrangements have been reported in papillary thyroid carcinoma (PTC) [4], in lung cancer [5], and chronic myelomonocytic leukemia [6]. Finally, overexpression of the *RET* gene has been demonstrated in the most aggressive estrogen receptor-positive breast cancer [7] and in the more advanced forms of pancreatic [8] and prostate cancer [9].

The *RET* proto-oncogene is subjected to alternative splicing that gives origin to three functional isoforms: *RET51*, *RET9*, and *RET43* [10,11]. Studies on animal models showed that *RET9* is expressed in several human tissues while *RET51* is only expressed in some of them [12]. Moreover, when compared, *RET9* expression has been found to be higher than *RET51* expression [13]. Conversely, *RET51* isoform expression has been reported to be higher in MTC than in PTC [14], in more aggressive forms of pancreatic cancer [15], and in pheochromocytoma [16], suggesting a specific role of this isoform in determining the aggressiveness of a tumor. As a matter of fact, the two isoforms are characterized by different biochemical and biological properties, and, consequently, they play distinct roles in tumorigenesis and/or development [17].

MTC arises from thyroid parafollicular C cells. Its overall incidence is about 0.2–0.8/100,000 people [18] and accounts for about 5% of all thyroid carcinomas. MTC can be inherited (25%) as part of the multiple endocrine neoplasia type 2, or sporadic (75%). The pathogenesis of this tumor is related to activating *RET* mutations that are germline in hereditary cases (approximately 98% of cases) and somatic in sporadic cases (approximately 45% of cases) [19,20]. The genetic landscape of sporadic MTC has been deeply studied, and somatic mutations in the *RET* gene are the major events in its tumorigenesis accounting for up to 80% of cases [21,22,23]. *H-RAS* (Harvey rat sarcoma virus) and *K-RAS* (Kirsten rat sarcoma virus) mutations are indeed present in about 10–20% of cases and are almost invariably mutually exclusive with *RET* mutations [21,24]. Only other rare genetic alterations have been reported, thus 20–40% of MTC cases are still orphans of a driver mutation [21,22,23].

The primary objective of the present study was to investigate whether *RET* gene over-expression, a mechanism of *RET* activation different from activating mutation, could be considered an alternative driver event for the development of MTC. As a secondary objective, we also evaluated the expression levels of the two *RET* isoforms (*RET9* and *RET51*) according to the mutational profile.

## 2. Materials and Methods

### 2.1. Patients

Eighty-three MTC patients were included in this study. Tissues were collected at surgery, immediately frozen in liquid nitrogen, and stored at −80 °C. All samples were previously analyzed by next-generation sequencing (NGS) IONS5, as previously described [21], and the mutational profile of our 83 cases was used to define the groups to be analyzed in the present study. In particular, we distinguished the 3 groups: cases with a somatic *RET* mutation (*RET*+), cases with a somatic *RAS* mutation (*RAS*+), and cases that were negative for *RET* and *RAS* mutations (*RET*− and *RAS*−).

All patients gave their consent to the study that was also approved by the Internal Reviewing Board.

### 2.2. Expression Analysis

RNA was extracted from fresh tissues using the TRIzol reagent lysis buffer (Invitrogen, Carlsbad, CA, USA) according to the protocol suggested by the manufacturer. Total RNA was quantified using the Qubit RNA HS Assay. cDNA was obtained by reverse transcription using the SuperScript IV VILO and 100 ng of total RNA in a final volume of 20 μL. The amplification of a house-keeping gene (Glucose-6-Phospate-dehydrogenase, G6PD) was used to verify the quality of cDNA.

To analyze the *RET* gene and *RET* gene isoform expression levels, we used the quantitative reverse transcription-polymerase chain reaction (qRT-PCR) technique with SsoAdvanced SYBR Green Supermix (Bio-Rad, Hercules, CA, USA). All reactions were performed with the BioRad CFX96 instrument.

Primers for the quantitative amplification of the *RET* tyrosine kinase domain were designed using the Primer3 software: (forward 5′ -> 3′ AACATCCTGGTAGCTGAGGG and reverse 5′ -> 3′ CAGCAGGACACCAAAAGACC) and were respectively located on exons 15 and 17. The efficiency and reproducibility of the primers were tested by a standard curve using a serial dilution of TT cDNA. The efficiency of the *RET TK* assay was E = 97.3%, R^2^ was 0.996, and the slope was −3.38. *RET9* and *RET51* isoforms were amplified using primers and conditions previously reported [14]. qRT-PCR reactions were performed in duplicate. Ct values of the replicates were similar (difference ≤ 0.5).

The *G6PD* housekeeping gene was used to normalize the *RET* gene expression level and its isoforms. It is not easy to find a reference tissue for MTC because normal thyroid tissue is not a counterpart of MTC, so 2-ΔΔCt is not applicable. This is the reason for which we decided to use the ΔCt analysis, a well recognized method for the analysis of the relative expression of genes. Gene expression level was calculated with the ΔCt method (ΔCt = Ct *RET* − Ct *G6PD*), where Ct is the threshold cycle for qRT-PCR. The lowest is the ΔCt value, and the highest is the mRNA expression level.

### 2.3. Statistical Analysis

Statistical analyses were performed with the Statview 5.0 Program using the Chi-squared test, 1-way ANOVA, and unpaired Student’s *t*-test. Differences were considered statistically significant when the *p*-value was less than 0.05.

## 3. Results

### 3.1. RET Gene Expression

**Figure 1:**
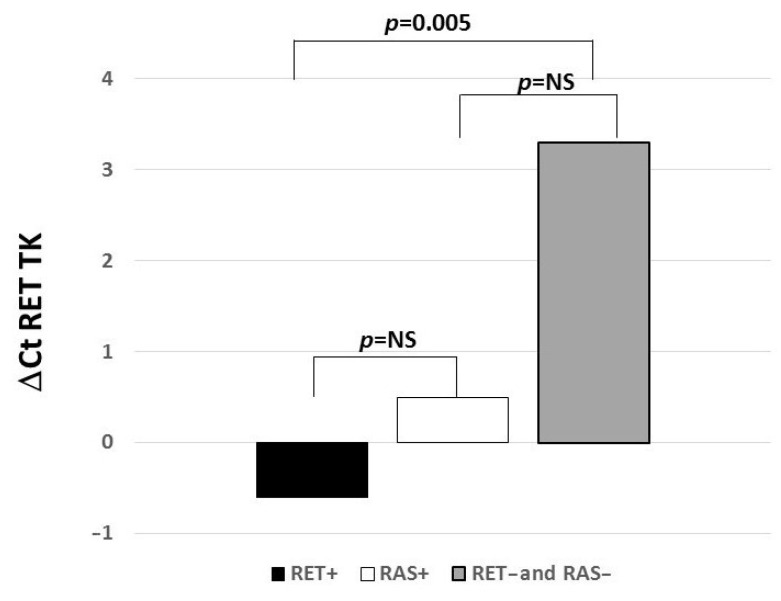
*RET* gene expression levels according to the somatic mutation profile. A statistically significant higher expression level was observed in *RET*+. Gene expression levels are reported as ΔCt (ΔCt = Ct *RET* − Ct *G6PD*) where Ct is the threshold cycle for qRT-PCR. The lowest is the ΔCt value, and the highest is the mRNA expression level.

Eighty-three samples previously analyzed by NGS for their mutation profile [21] were analyzed by qRT-PCR for *RET* gene expression. According to those data, cases with a *RET* somatic mutation (*RET*+, n = 39), cases with a *RAS* somatic mutation (*RAS*+, n = 20) and cases with no *RET* or *RAS* mutation (*RET*− or *RAS*−, n = 24). *RET* gene expression was detected in all cases regardless of the presence or absence of the mutations, although at different levels. As shown in Figure 1, a statistically significant higher expression of the *RET* gene was found in *RET*+ cases than in *RET*− and *RAS*− cases (*p* = 0.005). Although not statistically significant, *RET* gene expression was found to be higher in *RET*+ cases with respect to *RAS*+ cases. Similarly, *RAS*+ cases had a higher level of *RET* gene expression with respect to *RET*− and *RAS*− cases.

**Figure 2:**
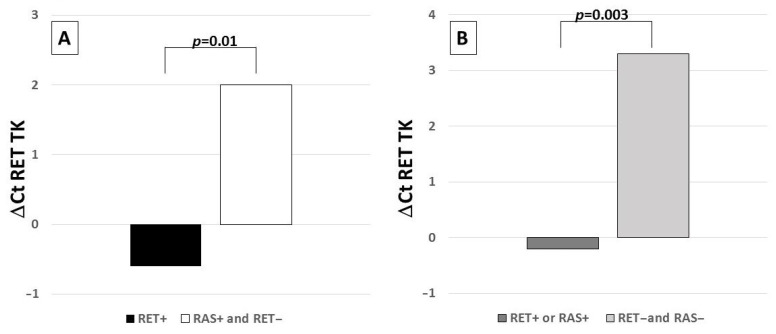
(**A**): *RET* gene expression levels according to the somatic mutation profile. A statistically significant higher *RET* gene expression level was observed in *RET*+ cases with respect to *RET*− and *RAS*+ cases. (**B**): gene expression levels according to the somatic mutation profile. A statistically significant higher *RET* gene expression level was observed in *RET*+ or *RAS*+ cases with respect to cases negative for both gene alterations (*RET*− and *RAS*−).

*RET* gene expression levels were found to be significantly higher in *RET*+ cases with respect to all other cases (i.e., *RET*− and *RAS*+) (*p* = 0.003) (Figure 2A). Similarly, cases positive for *RET* or *RAS* (*RET*+ or *RAS*+) showed a higher level of *RET* gene expression with respect to cases negative for both mutations (*RET*− and *RAS*−) (*p* = 0.01) (Figure 2B).

Expression levels are reported as ΔCt (ΔCt = Ct *RET* − Ct *G6PD*) where Ct is the threshold cycle for qRT-PCR. The lowest is the ΔCt value, and the highest is the gene expression level.

### 3.2. RET51 and RET9 Expression

As shown in Table 1, all cases (83/83, 100%) showed a *RET51* isoform expression while only 72/83 (86.7%) showed a *RET9* isoform expression. In the whole series, *RET51* expression levels were significantly higher (*p* < 0.0001) than *RET9* expression levels.

**Figure 3:**
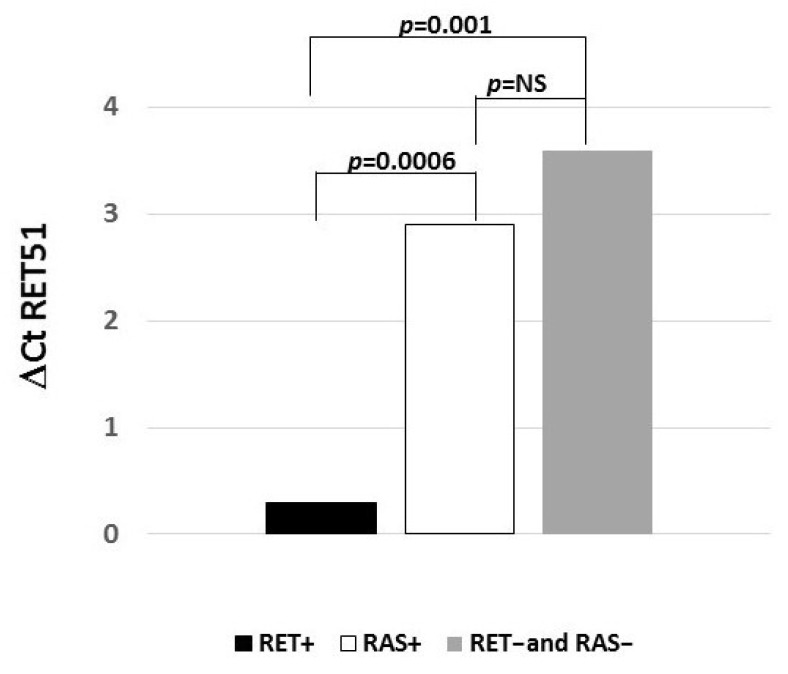
*RET51* isoform expression levels according to the somatic mutation profile. A statistically significant higher expression level was observed in *RET*+ cases than in *RAS*+ cases and *RET*− and *RAS*− cases. Gene expression levels are reported as ΔCt (ΔCt = Ct *RET* – Ct *G6PD*) where Ct is the threshold cycle for qRT-PCR. The lowest is the ΔCt value, and the highest is the mRNA expression level.

As shown in Figure 3, a statistically significant higher expression of the *RET51* isoform was found in *RET*+ cases than in *RAS*+ cases (*p* = 0.0006) and *RET*− and *RAS*− cases (*p* = 0.001). No difference in the *RET51* isoform expression levels was found when comparing *RAS*+ cases and *RET*− and *RAS*− cases.

**Figure 4:**
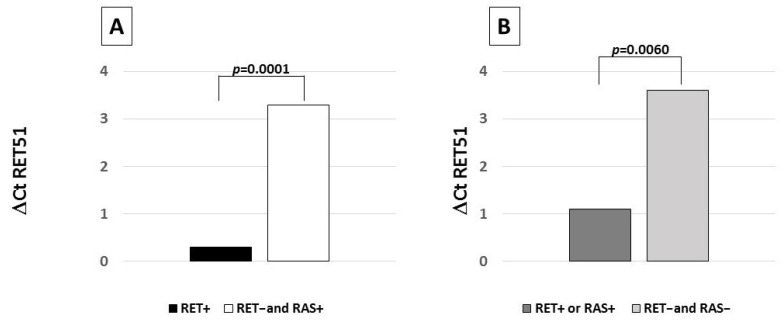
(**A**): *RET51* isoform expression levels according to the somatic mutation profile. A statistically significant higher *RET51* expression level was observed in *RET*-positive cases with respect to cases negative for a *RET* mutation. (**B**): *RET51* isoform expression levels according to the somatic mutation profile. A statistically significant higher *RET* gene expression level was observed in positive cases with respect to cases negative for both genes. Expression levels are reported as ΔCt (ΔCt = Ct *RET* – Ct *G6PD*) where Ct is the threshold cycle for qRT-PCR. The lowest is the ΔCt value, and the highest are the gene expression levels.

As shown in Figure 4A, a statistically significant higher expression of the *RET51* isoform was found in *RET*+ cases than in *RET*− and *RAS*+ cases (*p* = 0.0001). *RET51* expression levels were found to be significantly higher in *RET*+ and *RAS*+ positive cases with respect to *RET*− and *RAS*− cases (*p* = 0.006) (Figure 4B). The *RET9* isoform expression levels were not different in *RET*+, *RAS*+, and *RET*− and *RAS*− MTC cases.

## 4. Discussion

The *RET* proto-oncogene encodes for a tyrosine kinase receptor involved in the control of cell proliferation and differentiation [4]. Oncogenic mutations that constitutively activate the ret receptor have been reported in human tumors. *RET* mutations involved in the tumorigenesis processes can be classified as activating gain of function mutations, mainly in sporadic and hereditary MTC [25,26], and as gene rearrangements in PTC [26,27]. In addition to these alterations, increased expression of the wildtype *RET* is involved in the tumorigenesis and progression of some human tumors such as breast [7], pancreas [8], and prostate [9]. Recently, a higher *RET* gene expression has been observed also in MTC with respect to normal subjects [28]. In this study, we evaluated if an alternative mechanism of *RET* activation, such as overexpression, could be involved in MTC tumorigenesis, particularly in those cases that are still orphans of a driver mutation.

Using a qRT-PCR approach we demonstrated that *RET* gene expression levels are higher in MTC cases harboring a *RET* somatic mutation than in cases with a *RAS* somatic mutation or MTC cases without any of these mutations. These findings indicate that the increased rate of *RET* transcription and its higher expression cannot be considered as causative in *RET*− and *RAS*− cases. We recently demonstrated [29] that the prevalence of *RET* somatic mutations is higher in MTC of a larger tumor size, suggesting that the presence of this genetic alteration can induce a higher cell proliferation rate. According to these data, *RET*-mutated MTC cells could have a more active metabolic condition, thus also justifying higher levels of transcription. We could hypothesize that the over expression of *RET* mRNA can potentiate the transforming acitivity of *RET* mutations, thus concurring to the worse outcome of *RET*-mutated cases with respect to *RET*-negative cases and *RAS*-positive cases. *RET*-mutated tumors (both germline and somatic) show higher transcript levels of many epigenetic regulators than both *RET* wildtype and *RAS*-mutated MTC [30], and we can hypothesize that the higher *RET* gene expression could be due to the same mechanism.

Alternative splicing of the RET gene at the 3′ end has been described to produce two major isoforms: RET9 and RET51 [10,11]. A RET43 isoform has also been reported, although no evidence of its translational protein product has been provided so far [11]. The two isoforms are characterized by different biochemical and biological properties, and, consequently, they play distinct roles in tumorigenesis and/or development [17]. In particular, RET51 more effectively enhances cell proliferation and motility as well as maintains a more mesenchymal phenotype than RET9 [12,13] and is characterized by greater transforming potential [17,18]. In addition, previous studies using RET gene overexpression models have shown that RET51 has a greater transforming potential compared to RET9 [31,32,33]. In keeping with these observations, RET51 isoform expression is higher in MTC than in PTC [14] and in the more aggressive forms of pancreatic cancer [15], suggesting a specific role of this isoform in determining the aggressiveness of a tumor. In the present series, we evaluated the expression of the two RET isoforms, and we correlated the expression levels with the mutation profile. Interestingly, we found that all investigated cases were positive for RET51 expression while RET9 expression was found in 72/83 MTC cases, thus suggesting a predominant role of the longer isoform in MTC tumorigenesis. This predominant role has been confirmed by the observation that RET51 expression levels are higher than that of RET9.

We previously observed that overall *RET51* isoform is more expressed than *RET9* in MTC [14]. In the present study we demonstrated that *RET51* isoform expression is higher in MTC cases harboring a *RET* somatic mutation with respect to cases with either a *RAS* somatic mutation (i.e., *RAS*+) or any somatic mutation (i.e., *RET*− and *RAS*−). At variance, no different levels of expression were found when analysing *RET9* isoform expression. As reported by Le Hiret al. [16] it is likely that, in tumors caused by *RET* mutations, the presence of higher amounts of the long isoform can confer a selective growth advantage as demonstrated by the evidence that, in PC12 cells, the *RET51* isoform displays a more prominent potential as compared to the corresponding *RET9* isoforms [31,34]. This is in line with our previous evidence that sporadic MTC cases with a *RET* somatic mutation show a more rapid growth rate with respect to not-mutated cases in which *RET51* isoform is less expressed [29].

## 5. Conclusions

In conclusion, according to our data, *RET* gene over-expression does not play a role in MTC tumorigenesis, neither as an entire gene or as isoforms. At variance, *RET* gene, and in particular the *RET51* isoform, is expressed higher in *RET*-mutated cases. Taking into consideration that the *RET51* isoform seems to be able to confer a selective growth advantage, our previous results, showing that *RET* mutated cases have a high percentage of the mutated allele and that the corresponding tumors are usually bigger than not-mutated cases, are further supported. Moreover, the overexpression of *RET* could potentiate the transforming activity of mutated *RET*, making these cases more aggressive.

## Figures and Tables

**Table 1:** *RET9* and *RET51* isoforms expression in our series of 83 MTC cases.

*RET* Gene Expression	*RET9* n(%)	*RET51* n(%)	*p*
Pos expression	72 (86.7)	83 (100)	Not applicable
Neg expression	11	0	
ΔCt *	4.9	1.8	<0.0001

* Expression levels are reported as ΔCt (ΔCt = Ct *RET* – Ct *G6PD*) where Ct is the threshold cycle for qRT-PCR. The lowest is the ΔCt value, and the highest is the mRNA expression level.

## Data Availability

Data available on request due to restrictions, e.g., privacy or ethical.
